# Ozonated Olive Oil: Enhanced Cutaneous Delivery via Niosomal Nanovesicles for Melanoma Treatment

**DOI:** 10.3390/antiox11071318

**Published:** 2022-07-04

**Authors:** Sherif Ashraf Fahmy, Asmaa Ramzy, Amany M. Sawy, Mohamed Nabil, Mohamed Z. Gad, Mohamed El-Shazly, Mourad A. M. Aboul-Soud, Hassan Mohamed El-Said Azzazy

**Affiliations:** 1 Department of Chemistry, School of Sciences & Engineering, The American University in Cairo, AUC Avenue, P.O. Box 74, New Cairo 11835, Egypt; sheriffahmy@aucegypt.edu (S.A.F.); asmaaramzy95@aucegypt.edu (A.R.); amany.sawy@aucegypt.edu (A.M.S.); mohamed_nabeel@aucegypt.edu (M.N.); 2 School of Life and Medical Sciences, University of Hertfordshire Hosted by Global Academic Foundation, R5 New Garden City, New Administrative Capital, AL109AB, Cairo 11835, Egypt; 3 Department of Physics, Faculty of Science, Fayoum University, Fayoum 63514, Egypt; 4 Biochemistry Department, Faculty of Pharmacy and Biotechnology, The German University in Cairo, Cairo 11835, Egypt; mohamed.gad@guc.edu.eg; 5 Pharmacognosy Department, Faculty of Pharmacy, Ain-Shams University, Organization of African Unity Street, Abassia, Cairo 11566, Egypt; mohamed.elshazly@pharma.asu.edu.eg; 6 Pharmaceutical Biology Department, Faculty of Pharmacy and Biotechnology, The German University in Cairo, Cairo 11835, Egypt; 7 Chair of Medical and Molecular Genetics Research, Department of Clinical Laboratory Sciences, College of Applied Medical Sciences, King Saud University, P.O. Box 10219, Riyadh 11433, Saudi Arabia; maboulsoud@ksu.edu.sa; 8 Biochemistry Department, Cairo University Research Park, Cairo University, Giza 12613, Egypt

**Keywords:** ozone, olive oil, gas chromatography, niosomes, skin permeation, cancer therapy, melanoma

## Abstract

Ozonated olive oil (OL) combines the therapeutic effects of both ozone and olive oil. However, it suffers from limited water solubility and poor transdermal permeation, which hinder its application in melanoma treatment. Nanocarrier host molecules, such as niosomes, were used to improve the water solubility, transdermal permeation, and anticancer effect of hydrophobic compounds. This study aims to design and optimize a niosomal vesicular nanoplatform loaded with OL (OL/NSs) to improve OL’s skin permeation and anti-melanoma effect. In this regard, OL was prepared and characterized by evaluating its chemical properties (acid, peroxide, and iodine values) and fatty acid composition using gas chromatography. Then, OL/NSs were developed using the thin film hydration method employing cholesterol, Span 60, and Tween 60 at five different molar ratios. The optimized niosomes had an average diameter of 125.34 ± 13.29 nm, a surface charge of −11.34 ± 4.71 mV, and a spherical shape. They could entrap 87.30 ± 4.95% of the OL. OL/NSs showed a 75% sustained oil release over 24 h. The skin permeation percentage of OL/NSs was 36.78 ± 3.31 and 53.44 ± 6.41% at 12 and 24 h, respectively, three times higher than that of the free OL (11.50 ± 1.3 and 17.24 ± 2.06%, at 12 and 24 h, respectively). Additionally, the anticancer activity of the developed niosmal formulation, when tested on human melanoma cells (A375), was double that of the free OL; the IC_50_ of the OL/NSs was 8.63 ± 2.8 μg/mL, and that of the free OL was 17.4 ± 3.7 μg/mL. In conclusion, the encapsulation of ozonated olive oil in niosomes enhanced its water solubility, skin permeation, and anticancer activity and thus may represent potent natural chemotherapy in treating melanoma.

## 1. Introduction

Advanced-stage melanoma is responsible for most skin cancer deaths. However, the survival rates could exceed 90% if treated in the early stage [1]. Several synthetic chemotherapeutics are used to treat skin cancers, including alkylating agents, platinum-based drugs, and microtubule disrupting drugs. However, chemotherapeutics suffer from several shortcomings, including systemic toxic effects, allergy, and reduced bioavailability [2]. 

Natural products and oils contain several compounds that could serve as promising alternatives to synthetic chemotherapeutics [3,4,5]. Several studies have reported the anticancer activities of olive oil against colon [6,7], breast [8], ovarian [9], pancreatic [10], and lung [11] cancers. The anticancer activity of olive oil is attributed to its high content of polyphenolic compounds (mainly hydroxytyrosol and 3,4-Dihydroxyphenylethanol) and monounsaturated free fatty acids (such as linoleic, palmitic, arachidic, and oleic acids) [7]. 

Ozone has antimicrobial, wound healing, anti-inflammatory, and anticancer properties [12]. In addition, it is biocompatible with the skin and does not cause skin or mucous membrane irritation [12]. However, due to its high instability, ozone has been retained in natural oils containing high amounts of saturated and unsaturated fatty acids, such as olive oil. In this regard, ozonated olive oil (OL) has been produced, which combines the biological activities of both ozone and olive oil [13]. It is also of note that OL is commercially available as a skincare product (for acne, wrinkles, rash, and eczema) [13].

Despite its promising biological applications, to date, there are no studies on using OL in skin cancer treatment. This could be attributable to its limited water solubility and limited transdermal permeation.

Niosomes are amphiphilic nanostructures, comprised mainly of cholesterol and nonionic surfactants, and are prepared by self-assembly of the latter in an aqueous medium, forming bilayer closed nanovesicles [14]. Niosomes are fabricated using sonication, microfluidization, or thin-film hydration techniques. However, the latter technique is widely used for its simplicity, followed by sonication to obtain niosomes of homogenous particle sizes [14]. Compared to other nanovesicles (such as liposomes and polymersomes), niosomes are cheaper, easier to design, and more stable when stored at 4 °C for up to 6 months [15]. Using niosomes in skin drug delivery is very promising because they are inert, biocompatible, biodegradable, and can accommodate hydrophobic and hydrophilic drugs [14]. In addition, they can improve skin permeation and therapeutic efficiency and enable controlled drug release [15]. Furthermore, niosomes can increase the residence time of various drugs in the stratum corneum and epidermis, while minimizing their systemic absorption [16]. Several studies have reported the encapsulation of different drugs into niosomes, such as topotecan [14], letrozole [17], curcumin [18], and melittin [19], aiming to improve their hydrophilicity, bioavailability, and anticancer activities.

Thus, in the present study, we designed ozonated olive oil-loaded niosomes (OL/NSs) and explored their potential in treating melanoma. In this regard, OL was prepared and chemically characterized by estimating its acid, peroxide, and iodine values, and its composition was evaluated using gas chromatography. Then, OL/NSs were developed using the thin film hydration method employing cholesterol, Span 60, and Tween 60 at five different molar ratios. The optimized formula was selected based on average diameter, PDI, zeta potential, and entrapment efficiency (EE%). Then the optimized OL/NSs were subjected to further investigations in terms of morphology, release, skin permeation percentage, and anticancer activity against melanoma cells.

## 2. Materials and Methods

### 2.1. Materials

Cholesterol, Span 60, and Tween 60 were obtained from Biosynth Carbosynth, Berkshire, UK. Dulbecco’s modified Eagle’s medium (DMEM) with 4.5 g/L glucose, 0.05% Trypsin and phosphate-buffered saline (PBS, pH 7.4), Penicillin-streptomycin, and 3-(4,5-Dimethylthiazol-2-yl)-2,5 diphenyltetrazolium bromide (MTT) were purchased from Lonza Bioscience (Walkersville, MD, USA). Fetal bovine serum (FBS) was obtained from Gibco (Waltham, MA, USA). Dimethyl sulfoxide (DMSO) was obtained from Serva (Heidelberg, Germany). All other chemicals were purchased from Sigma-Aldrich, St. Louis, MO, USA.

### 2.2. Preparation of Ozonated Olive Oil (OL)

Ozone gas was generated using a 4G lab benchtop ozone generator (Model: A2ZS, A2Z Ozone^®^, Louisville, KY, USA) equipped with an oxygen tank (purity > 99%). Italian olive oil (1 L) was poured into a 2 L Drechsel bottle and placed in a cooling bath at −2 °C. The ozone at 4 g/h was bubbled in the olive oil for 36 h until the oil color became white and the consistency semisolid. The formed, ozonated olive oil was scraped from the Drechsel bottle, placed in a glass jar, weighed, and stored at 4 °C.

### 2.3. Characterization of OL

#### 2.3.1. Determination of Acid, Peroxide, and Iodine Values

The acid, peroxide, and iodine values were determined by following AOAC official analysis methods [20].

To determine the acid value (AV), an adequate amount of alcohol was heated with 0.5 mL of phenolphthalein until boiling and neutralized by NaOH (0.1 N). Then, 50 mL of the hot neutralized alcohol was added to a 20 g OL sample, then boiled. The solution was then titrated with NaOH (0.1 N) with vigorous shaking. The acid value was calculated using Equation (1).

(1) $$\mathrm{Acid}\;\mathrm{value}=\frac{56.1\;*\;\mathrm{Vs}\;*\;N}{W}$$

where Vs is the volume of 0.1 N NaOH, N is the normality of NaOH, and W is the weight of the OL sample.

The peroxide value (PV) was determined by dissolving 5 g of OL sample in a mixture of glacial acetic acid to chloroform (1:1). Then, 0.5 mL of saturated potassium iodide solution was added, mixed, and incubated in the dark for 10 min. After this, 30 mL of distilled water was added, and the solution was titrated with 0.1 M sodium thiosulphate solution in a stoppered conical flask. The same steps were followed for the blank solution. The peroxide value was determined utilizing Equation (2).

(2) $$\mathrm{Peroxide}\;\mathrm{value}\;\left(\mathrm{PV}\right)=\frac{M\;*\;100\;*\;\left(\mathrm{Vs}-\mathrm{Vb}\right)}{W}$$

where M is the molarity of sodium thiosulphate solution, Vs is the volume of the OL sample, Vb is the blank volume, and W is the weight of the OL sample.

The iodine value (IV) was determined by dissolving 0.32 g of OL sample in a 15 mL mixture of 1:1 cyclohexene-acetic acid solution and Wijs solution (25 mL). The mixture was kept in the dark for 60 min in a stoppered conical flask. Then the solution was titrated with 0.1 M sodium thiosulphate solution after adding 20 mL of potassium iodide solution and 150 mL of distilled water. The same steps were followed for the blank solution.

The iodine number (IV) was estimated using Equation (3).

(3) $$\mathrm{Iodine}\;\mathrm{value}=\frac{\left(B-S\right)\;*\;M\;*\;12.69}{W}$$

where B is the blank volume, S is the volume of the OL sample, M is the molarity of sodium thiosulphate solution, and W is the weight of the OL sample.

#### 2.3.2. Gas Chromatography (GC) Analysis

Esterification of the OL sample was conducted before gas chromatography analysis. Briefly, 2 mL of isooctane was added to a 0.1 g OL sample, followed by 0.1 mL of 2 M methanolic potassium hydroxide solution. After vigorous shaking, 2 mL of 0.1 M NaCl solution was added, then the organic layer was separated and transferred to the sample vial. Finally, 1 g of sodium hydrogen sulfate was added to the sample vial, mixed, and the solution was ready for GC analysis.

GC analysis was carried out using an autosampler Clarus 580 (PerkinElmer, Norwalk, CT, USA), Rt-560 capillary column (100 m × 0.25 mm × 0.20 µm; Restek, Bellefonte, PA, USA), and flame-ionization detection (FID). Chromatography software (Totalchrom 6.3.2, PerkinElmer, Norwalk, CT, USA) was used for data acquisition from the FID. Helium was used as the carrier gas with split injection (100:1). The analyses were carried out in programmed temperature mode from 100 to 240 °C and then isothermal for 15 min. The detector temperature was 250 °C, and the injector temperature was 240 °C. The results were expressed as a percentage of individual fatty acids in lipid fractions.

The analysis was repeated twice in the following sequence: blank (isooctane solvent), fatty acid methyl ester (FAME) mixture standard, internal QC sample, ozonated olive oil sample, repeated FAME mixture standard, repeated internal QC sample, and repeated ozonated olive oil sample.

### 2.4. Preparation and Optimization of Ozonated Olive Oil Loaded Niosomes (OL/NSs)

OL/NSs were prepared by the thin-film hydration technique [21] with some modifications. A total of 120 mmol of cholesterol, Span 60, and Tween 60 were used in five different molar ratios (2:1:1, 2:1.5:1, 2:2:1, 2:2.5:1, and 2:3:1, respectively). Briefly, the cholesterol, surfactants, and OL were dissolved in a chloroform/diethyl ether mixture (1:1, *v/v*). The organic solvent was evaporated under reduced pressure for 1 h at 60 °C, utilizing a Laborota 4000 rotary evaporator (Heidolph Instruments, Schwabach, Germany) equipped with a vacuum pump (KNF Neuberger GmbH, Freiburg, Germany), leaving a thin lipid film. The thin film was hydrated in phosphate buffer saline (pH 7.4) in a rotary evaporator under normal pressure at 60 °C for 1 h. The obtained suspensions were sonicated for 2 min using a bath sonicator (Elmasonic P30 H, Elma Hans Schmidbauer, Singen, Germany). The prepared suspensions were left for 45 min at room temperature and then kept at 4 °C for further investigation.

### 2.5. Characterization of the Prepared OL/NSs

The average particle size, PDI, and zeta-potential were studied employing a Zetasizer Nano ZS equipped with a 10 mW HeNe laser (Malvern Instruments, Worcestershire, UK). The size measurement was conducted in triplicate at 25 °C.

The morphology of the OL/NSs was examined utilizing transmission electron microscopy (TEM) (JEOL-JEM 2100 electron microscope, Musashino, Akishima, Tokyo, Japan) operating at 160 kV.

The FTIR spectral data (4000−500 cm^−1^) were obtained using an FTIR-8400s instrument (Shimadzu, Kyoto, Japan). Samples were compressed with KBr under hydraulic pressure, then analyzed.

### 2.6. Entrapment Efficiency (EE%) of OL in Niosomes

The EE% of the loaded OL was determined by removing the free OL via centrifugation at 16,000 rpm and 4 °C for 3 h. Then, the supernatant was separated, and the free OL was measured using a FLUOstar Omega microplate reader (BMG Labtech, Offenburg, Germany) UV-Vis spectrophotometer at 215 nm. The EE% was computed using Equation (4) [22].

(4) $$\mathrm{EE}\;\%=\frac{\mathrm{Initial}\;\mathrm{amount}\;\mathrm{of}\;\mathrm{drug}-\mathrm{the}\;\mathrm{amount}\;\mathrm{of}\;\mathrm{free}\;\mathrm{drug}}{\mathrm{Initial}\;\mathrm{amount}\;\mathrm{of}\;\mathrm{drug}}\times 100$$

### 2.7. In-Vitro Release Study

The release rates of OL from the OL/NSs were examined utilizing the dialysis membrane method, and the free OL was used as a control. A known amount of OL/NSs nanoparticles was placed in a dialysis bag (12–14 KD cut off) and placed in 50 mL of PBS (pH 5.5). The system was left in a shaking incubator (Jeio tech SI-300, Seoul, Korea), rotating at 300 rpm at 37 ± 1 °C. At certain time intervals, 1 mL aliquot of the buffer was pipetted and immediately replaced with the same volume of fresh buffer to maintain sink conditions. The released OL was determined by measuring its absorbance at 215 nm. The release % was calculated utilizing Equation (5).

(5) $$\mathrm{Release}\;\left(\%\right)=\frac{\mathrm{Amount}\;\mathrm{of}\;\mathrm{released}\;\mathrm{drug}}{\mathrm{Initial}\;\mathrm{amount}\;\mathrm{of}\;\mathrm{loaded}\;\mathrm{drug}}\times 100$$

### 2.8. Ex-Vivo Skin Permeation and Deposition Studies

#### 2.8.1. Skin Preparation

The back skin of Sprague Dawley male rats (220–240 g) was shaved using electrical clippers. The subcutaneous fats were separated from the skin utilizing a scalpel, washed with PBS saline, wrapped in aluminum foil, and stored at −20 °C for further use. Skin permeation and deposition tests were conducted employing Franz diffusion cells.

#### 2.8.2. Skin Permeation Test

A full-thickness piece of skin was mounted between donor and receptor chambers of the Franz-type diffusion cells (diffusion area was 1.23 cm^2^). The receptor compartments were filled with 80% PBS (pH 5.5) and a 20% organic mixture of acetonitrile: methanol: ethanol with a 2:1:1 ratio and kept at 37 ± 2 °C with constant stirring using a Teflon magnetic bar.

OL/NSs nanoparticles and free OL solution were applied to the donor chambers for the skin permeation evaluation. At 12 h and 24 h, sample aliquots (500 µL) were pipetted from the receptor compartment and replaced immediately with the same volume of fresh PBS. Samples were then quantified spectrophotometrically at 215 nm. The trials were conducted in triplicate, and the results were expressed as the mean value ± standard deviation.

### 2.9. Cell Viability Assessment

#### 2.9.1. Cell Culture

Human melanoma cells, A375, (American Type Culture Collection, Manassas, VA, USA) were grown in DMEM provided with 10% heat-inactivated fetal bovine serum (FBS) and 1% penicillin-streptomycin and maintained at 37 °C in a humidified atmosphere (5% CO_2_).

#### 2.9.2. MTT Assay

A375 cells were treated with different concentrations of OL, OL/NSs, and empty niosomes (NSs). An MTT assay examined the cell viability of cancerous cells, and the IC_50_ value (μg/mL) was calculated as described previously [23].

### 2.10. Statistical Analysis

All experiments were carried out in triplicate, and the standard deviations (SD) were computed. Statistical analysis was performed by GraphPad Prism software version 8.0 (GraphPad, San Diego, CA, USA) using a one-way ANOVA test, considering the difference statistically significant when the *p*-value was < 0.05.

## 3. Results and Discussion

### 3.1. Characterization of the OL

The acidity, peroxide, and iodine values of the ozonated olive oil were 8.07 mg KOH/g oil, 105.2 meq O_2_/Kg, and 44.8 g I_2_/100 g, respectively. The ozonated olive oil showed high acid and peroxide values, indicating its high oxidizing power potential. On the other hand, the low iodine value indicated the successful ozonation of olive oil as the addition of ozone decreased the number of double bonds [24,25].

**Figure 1:**
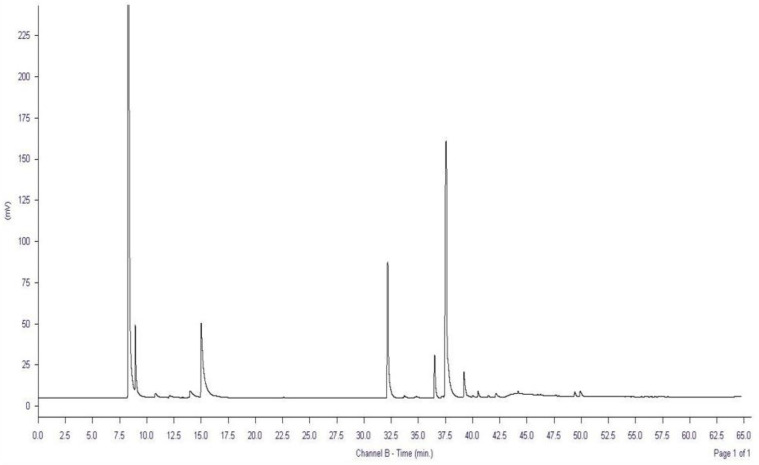
GC chromatogram of ozonated olive oil (OL).

Additionally, the fatty acid composition of the OL was investigated using gas chromatography (GC). Data presented in Table 1 and Figure 1 show that major fatty acids (oleic acid, the most abundant fatty acid in olive oil, palmitic and caprylic acids) responsible for the biological effects of olive oil are retained after ozonation. Since ozonation attacks mainly the double bonds of the unsaturated fatty acids, causing a reduction in their numbers [26], it is crucial to retain outstanding amounts of the major fatty acids after ozonation to ensure the combined biological activities of both ozone and olive oil.

### 3.2. Characterization of the OL loaded Niosomes (OL/NSs)

#### 3.2.1. Average Diameters, PDI, Zeta-Potential, and Entrapment Efficiency (EE%)

Different ratios of cholesterol (Ch), Span 60 (S60), and Tween 60 (T60) (2:1:1, 2:1.5:1, 2:2:1, 2:2.5:1, and 2:3:1, respectively) were used to design the niosomes loaded with OL. The best-optimized formulation with respect to size, PDI, zeta potential and EE% was selected for further experiments.

In this regard, the average diameters and PDI of the plain niosomes and different formulations were measured by dynamic light scattering (Table 2). OL/NSs prepared using Ch/S60/T60 in a ratio of 2:2:1 had the lowest average diameter (125.34 ± 13.29 nm), PDI (0.24 ± 0.04), and the highest zeta potential (−11.34 ± 4.71 mV), as compared to the rest of formulations.

In transdermal drug delivery, the carriers’ size is a critical factor that controls the penetration through the skin surface. Small particle sizes (below 200 nm) were reported to have enhanced skin permeation [27]. Additionally, small particle sizes are vital to improving the uptake of the drugs into the cancerous cells via passive targeting through the enhanced permeability and retention effect [28]. Moreover, a high negative charge would help prevent particle clustering and prolong the drug’s shelf-life stability [23].

Furthermore, it was shown that N3 had the highest EE% (87.30 ± 4.95%), relative to the other formulations. The enhanced EE% of N3 compared to other formulas is attributed to its smaller size and hence the higher surface area to volume ratio. This high EE% would result in a more significant anticancer effect.

Since the OL/NSs formula prepared using Ch/S60/T60 in a ratio of 2:2:1 showed the lowest average diameters and the highest ζ-potential and EE% compared to the other four preparations, it was chosen for further study investigations (OL/NSs).

#### 3.2.2. Morphological and Chemical Analyses of the Optimized OL Loaded Niosomes (OL/NSs)

**Figure 2:**
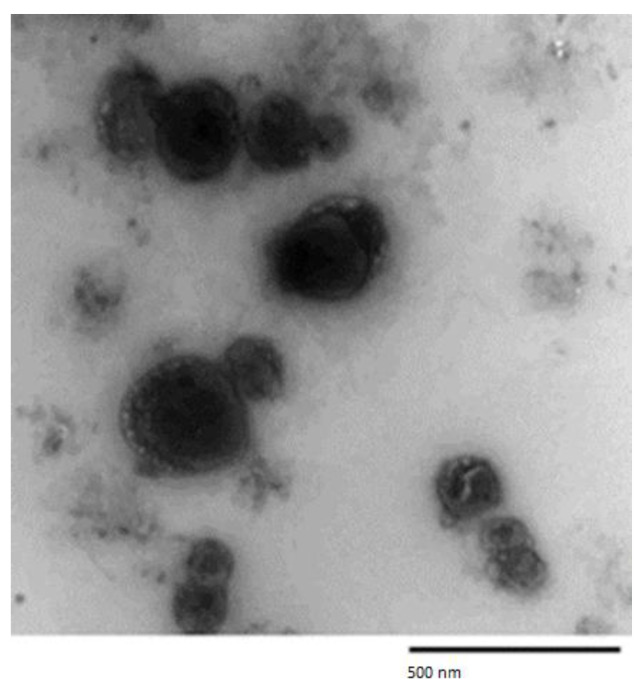
Transmission electron microscope (TEM) image of the optimized OL/NSs.

TEM analysis of the OL/NSs formulation showed a spherical shape and closed vesicular morphology (Figure 2).

**Figure 3:**
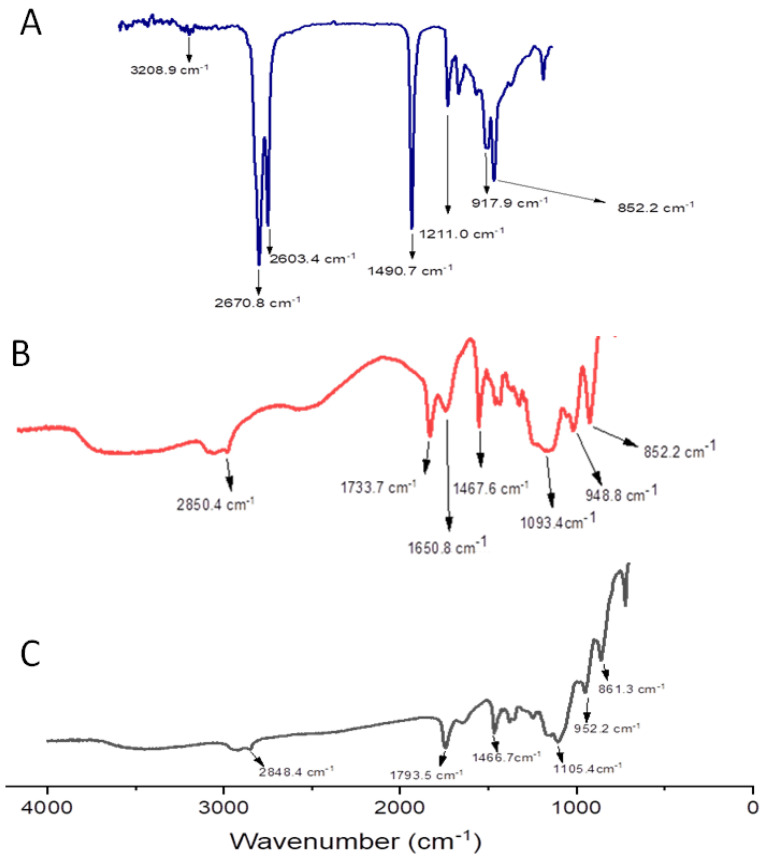
FTIR spectra of (**A**) free OL, (**B**) niosomes, and (**C**) OL/NSs.

In order to study the structural features of the prepared niosomes compared to free OL, the FTIR spectra of OL, empty niosomes, and OL/NSs were obtained (Figure 3).

The FTIR spectrum of ozonated olive oil (Figure 3A) showed four major peaks at 3208.9 cm^−1^, (OH stretching), 2670.8 cm^−1^ (C-H stretching), 1490.7 cm^−1^ (C-C stretching), and 1211.0 cm^−1^ (C-O stretching) [29]. The FTIR spectrum of plain niosomes (Figure 3B) showed four characteristic peaks at 2850.4 cm^−1^ (C-H stretching), 1650.8 cm^−1^ (C=O stretching), 1467.6 cm^−1^ (C-H bending), and 1093 cm^−1^ (C-O stretching) [30]. On the other hand, the FTIR spectra of OL/NSs (Figure 3C) demonstrated four major peaks at 2848.4 cm^−1^ (C-H stretching), 1793.5 cm^−1^ (C=O stretching), 1466.7 cm^−1^ (C-H bending), and 1105.4 cm^−1^ (C-O stretching). FTIR data showed the presence of all OL peaks in the OL/NSs, suggesting physical entrapment of the oil inside the niosomal matrix.

#### 3.2.3. In-Vitro Release Study

**Figure 4:**
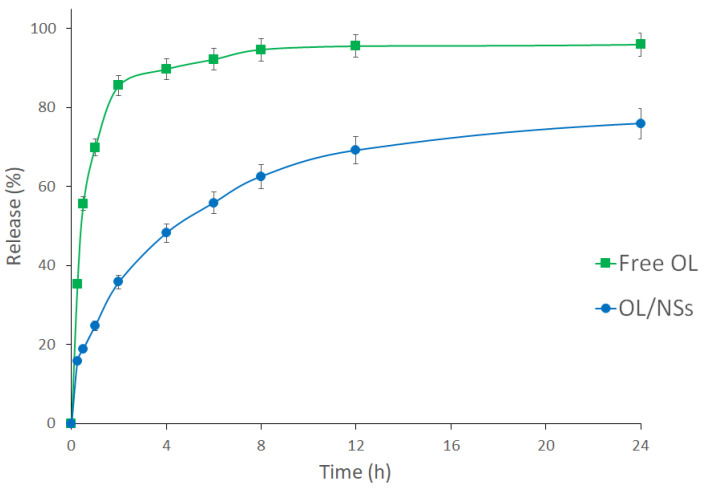
In vitro release profiles of free OL (square) and OL/NSs (circle) at 37 °C and pH 5.5 up to 24 h.

The in vitro drug release study was carried out to examine the release of the OL from the niosomal carrier at a pH of 5.5 and 37 ± 1 °C. Free OL was used as a control (Figure 4). In the case of free OL, almost all the loaded oil (about 90%) passed the dialysis membrane (cutoff 10 kDa) during the first 4 h. On the other hand, 45%, 69%, and 75% of the OL were released from the niosomal formulation during the first 4, 12, and 24 h, respectively. This shows that encapsulation of OL in NSs achieved a more sustained release behavior.

The sustained release of OL from OL/NSs is attributed to the cholesterol used in the synthesis of niosomes, which enhances the stability of the niosomal membrane and reduces the permeability of the entrapped OL across the niosomal membrane [31,32].

#### 3.2.4. Ex-Vivo Skin Permeation Study

**Figure 5:**
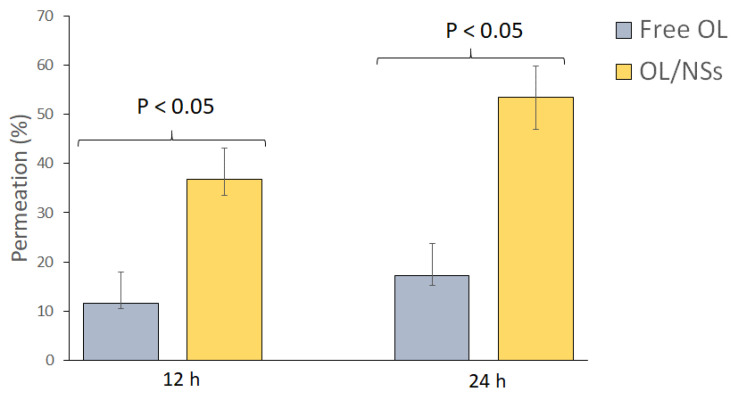
Ex-vivo skin permeation study of OL and OL/NSs.

The ex vivo permeation study was carried out to assess the impact of the niosomes as a nanocarrier on the permeation of OL across the skin layers. The skin permeation results of the free OL compared to OL/NSs at 12 and 24 h are presented in Figure 5. The skin permeation percentage of free OL was 11.50 ± 1.3 and 17.24 ± 2.06% at 12 and 24 h. On the other hand, the skin permeation percentage of OL/NSs was 36.78 ± 3.31 and 53.44 ± 6.41% at 12 and 24 h. The drug permeation in the case of OL/NSs is three times higher than that of the free oil (*p* < 0.05). These findings are in line with previous studies that reported an increase in the permeation of drugs after being loaded into vesicular nanocarriers [33,34]. The increased permeation in the case of niosomal formulations is attributed to the presence of nonionic surfactants (Span 60 and Tween 60), which serve as a permeation enhancer that could improve the drug penetration across skin layers [35]. Moreover, the nanosized niosomes can loosen the stratum corneum’s closely packed cellular structure by increasing its hydration [36].

#### 3.2.5. Cell Viability Assay

**Figure 6:**
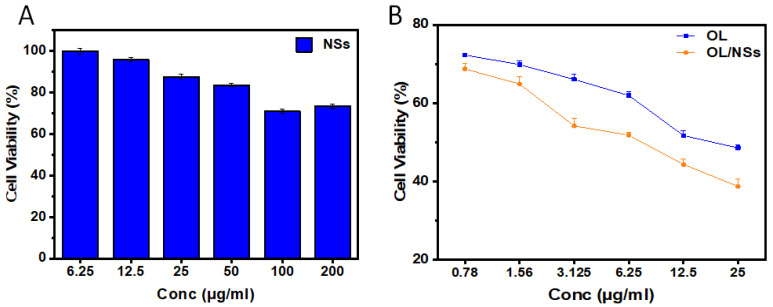
Evaluating the cytotoxicity of (**A**) plain niosomes, (**B**) OL, and OL/NSs on A375 cells at various concentrations using MTT assay. An overall statistically significant decrease in cell viability was observed with OL/NSs compared to free OL (*p* < 0.05). Untreated cells were used as negative control and considered as 100%. All experiments were carried out in triplicate, and the mean values were calculated. Error bars represent ± standard deviation.

The cytotoxic effects of plain NSs, OL, and OL/NSs were studied using MTT assay. To ensure that the cytotoxic effect of the niosomes loaded with OL is not attributed to the niosomes alone, we evaluated the effect of plain niosomes on A375 cellular viability (Figure 6A). None of the concentrations assessed of the plain NSs had any significant cytotoxicity on A375 cells, excluding the probability that the effect of OL/NSs is due to the cytotoxic effects of the NSs. On the other hand, OL or OL/NSs showed a high percentage of cell death in a dose-dependent manner (Figure 6B). The IC50 values of OL and OL/NSs were 17.4 ± 3.7 and 8.63 ± 2.8 μg/mL, respectively. It was evident that the cytotoxicity of OL/NSs was significantly (*p* < 0.05) higher than that of free OL by a 2-fold difference. The enhanced cytotoxicity of OL/NSs, as compared to OL, is attributed to the improvement of the hydrophilicity of OL after its encapsulation in the niosomal carriers. Additionally, the nanosized niosomal formulation can better diffuse into melanoma cells via the enhanced permeability and retention (EPR) effect [28]. Additionally, it has been reported that using a nonionic surfactant (Span 60) in designing niosomes would enhance the anticancer activity of the loaded drug [37]. These results support the promising use of niosomes as a potential carrier for various hydrophobic natural anticancer drugs in transdermal drug delivery.

## 4. Conclusions

In this work, ozonated olive oil was prepared to combine the therapeutic effects of both olive oil and ozone and loaded into a niosomal nanosystem. Five different niosomal formulas of cholesterol, Span 60 and Tween 60 were prepared and characterized. The niosomal formulation with the smallest size, PDI, and the highest surface charge and EE% was loaded with OL. The optimized OL/NSs achieved a sustained release behavior for OL and enhanced skin permeation as compared to free OL. In addition, OL/NSs exerted double the anticancer activity of free OL when tested on A375 cells. Considering the high anticancer activity and skin permeation of the OL/NSs as compared to free OL, niosomes made of Ch/S 60/T60 (2:2:1) and loaded with ozonated olive oil represent a promising formula for treating melanoma and cutaneous delivery of other natural hydrophobic compounds.

## Figures and Tables

**Table 1:** Fatty acids identified in the ozonated olive oil (OL) using GC.

Composition of Fatty Acids	Ozonated Olive Oil (OL)	
	Time (min)	(%)
Caproic acid C 6:0	10.781	0.84
Caprylic acid C 8:0	14.992	18.79
Palmitic acid C 16:0	32.194	19.75
Palmitoleic acid C 16:1	33.729	0.34
Margaric acid C 17:0	34.815	0.28
Stearic acid C 18:0	36.507	4.90
Oleic acid C 18:1	37.584	48.19
Linoleic acid C 18:2	39.202	3.59
Arachidic acid C 20:0	40.495	0.72
Linolenic acid C 18:3	41.452	0.20
Behenic acid C 22:0	44.183	1.54
Lignoceric acid C 24:0	47.673	0.10

**Table 2:** The average particle size, PDI, ζ-potential, and EE% of different niosomal formulas. All experiments were conducted in triplicate, and the results were expressed as means ± standard deviations.

Formula Code	Molar Ratio			Diameter (nm)	PDI	Zeta Potential (mV)	EE %
	Ch	S60	T60				
N1	2	1	1	388.27 ± 12.23	0.32 ± 0.14	−7.65 ± 2.63	0.90 ± 1.94
N2	2	1.5	1	223.07 ± 8.99	0.31 ± 0.13	−7.57 ± 2.97	17.05 ± 5.23
N3	2	2	1	125.34 ± 13.29	0.24 ± 0.04	−11.34 ± 4.71	87.30 ± 4.95
N4	2	2.5	1	356.38 ± 18.42	0.31 ± 0.08	−6.89 ± 5.78	8.76 ± 9.65
N5	2	3	1	342.66 ± 28.9	0.33 ± 0.11	−1.64 ± 1.44	44.55 ± 3.20

## Data Availability

Data are available in the manuscript.
